# A Case of Orbital Cellulitis in a Seven-Year-Old Girl: The Diagnostic Significance of Intraorbital Air on Contrast-Enhanced CT

**DOI:** 10.7759/cureus.81179

**Published:** 2025-03-25

**Authors:** Alice Kodama, Ryutaro Ohira, Shoichiro Kanda, Keiichi Takizawa, Akiko Kinumaki

**Affiliations:** 1 Pediatrics, The University of Tokyo, Tokyo, JPN

**Keywords:** chandler’s stages, intraorbital gas, medial rectus muscle, oribital cellulitis, perioribital cellulitis

## Abstract

Orbital and periorbital cellulitis are common pediatric infections requiring accurate differentiation, as orbital cellulitis can lead to severe complications if left untreated.

A seven-year-old girl with a history of group A β-hemolytic *Streptococcus *(GAS) infection six months earlier presented with fever and progressive periorbital swelling. Differentiation between orbital and periorbital cellulitis based on physical examination alone was challenging due to the absence of proptosis and ophthalmoplegia. Contrast-enhanced CT revealed left-sided sinusitis, fat stranding extending beyond the orbital septum, partial erosion of the lamina papyracea, and intraorbital gas, confirming orbital cellulitis. Broad-spectrum antibiotics, cefotaxime (300 mg/kg/day), clindamycin (900 mg/day), and vancomycin (1500 mg/day), were administered, leading to rapid clinical improvement. *Moraxella catarrhalis *was identified from nasal and ocular discharge cultures, and the patient fully recovered after three weeks of antibiotic therapy without surgical intervention.

This case highlights the importance of contrast-enhanced CT in distinguishing orbital from periorbital cellulitis, especially in cases lacking classical orbital findings. The presence of intraorbital gas served as a key diagnostic clue, emphasizing the need for early imaging in suspected orbital cellulitis to guide appropriate management. In particular, this case demonstrates that careful assessment of symptom presentation and clinical progression can support early consideration of orbital involvement and timely decision-making regarding CT imaging.

## Introduction

Orbital and periorbital cellulitis are common pediatric infections requiring accurate differentiation and management. Periorbital (preseptal) cellulitis is a soft tissue infection confined anterior to the orbital septum, causing eyelid erythema and swelling. In contrast, orbital cellulitis involves posterior spread, leading to proptosis, ophthalmoplegia, and visual impairment. While both respond to antibiotics, untreated orbital cellulitis may cause severe complications such as abscesses, meningitis, and cavernous sinus thrombosis. Early diagnosis and intervention are crucial.

Orbital cellulitis can be classified into five progressive stages according to Chandler’s classification, ranging from preseptal inflammation to life-threatening cavernous sinus thrombosis [1,2]. This staging helps clinicians assess severity and guide appropriate intervention.

Imaging studies, particularly contrast-enhanced CT, play a vital role in the early diagnosis of orbital cellulitis. Clinical guidelines, such as the *Guidelines for the Management of Periorbital Cellulitis/Abscess* [2], provide recommendations on when CT should be considered. These include signs such as proptosis, ophthalmoplegia, deteriorating vision, or lack of clinical improvement. However, in certain cases, careful attention to the clinical course and symptom progression may allow for early diagnosis even in the absence of classic signs, potentially avoiding the need for surgical intervention.

Orbital cellulitis mainly affects children, with 50% of cases occurring in those under six years of age and 80% in individuals under 20 [3]. Sinusitis, present in approximately 90% of cases, is the most common cause, followed by odontogenic and facial infections, otitis media, trauma, and postoperative complications [1,4]. Some reports suggest intraorbital gas, linked to anaerobic bacteria, as a diagnostic clue in sinusitis-associated cases [5-7]. Here, we present a school-aged child with orbital cellulitis in which intraorbital gas aided diagnosis. Early recognition of the clinical course led to timely imaging, enabling a prompt diagnosis and successful conservative management without surgery.

## Case presentation

The patient was a seven-year-and-five-month-old girl with no notable perinatal or developmental history. She had a history of group A β-hemolytic *Streptococcus* (GAS) infection six months prior. She did not exhibit accompanying symptoms such as nasal obstruction or nasal discharge.

On the morning prior to presentation, she developed a fever of 39.5°C and a headache. By that night, she began experiencing left retro-orbital pain and photophobia. Over time, she also developed progressive swelling around the left eyelid, which eventually made it difficult for her to open her left eye.

On Day 2 of illness, her body temperature reached 40.1°C. A nearby clinic’s rapid antigen tests on a nasopharyngeal swab were negative for influenza virus and SARS-CoV-2 (severe acute respiratory syndrome coronavirus 2). However, a rapid antigen test on a throat swab was positive for GAS. Given her prior positive GAS test six months earlier, asymptomatic carriage was considered. Conjunctival injection was observed in the left eye only. On the same day, ocular discharge appeared and progressively increased, accompanied by worsening swelling and redness of the left eye. As a result, hospitalization was deemed necessary, and she was referred to our institution.

In the absence of obvious proptosis or visual impairment, although not formally assessed using a Hertel exophthalmometer, periorbital cellulitis was initially considered. However, the time course of symptoms, including fever, headache, and the subsequent development of left retro-orbital pain and photophobia, raised suspicion for orbital cellulitis, especially given the rapid progression of periorbital swelling. Considering that orbital cellulitis is a severe infection with potential for significant complications and long-term sequelae, a contrast-enhanced head CT scan was performed on the day of admission.

**Figure 1:**
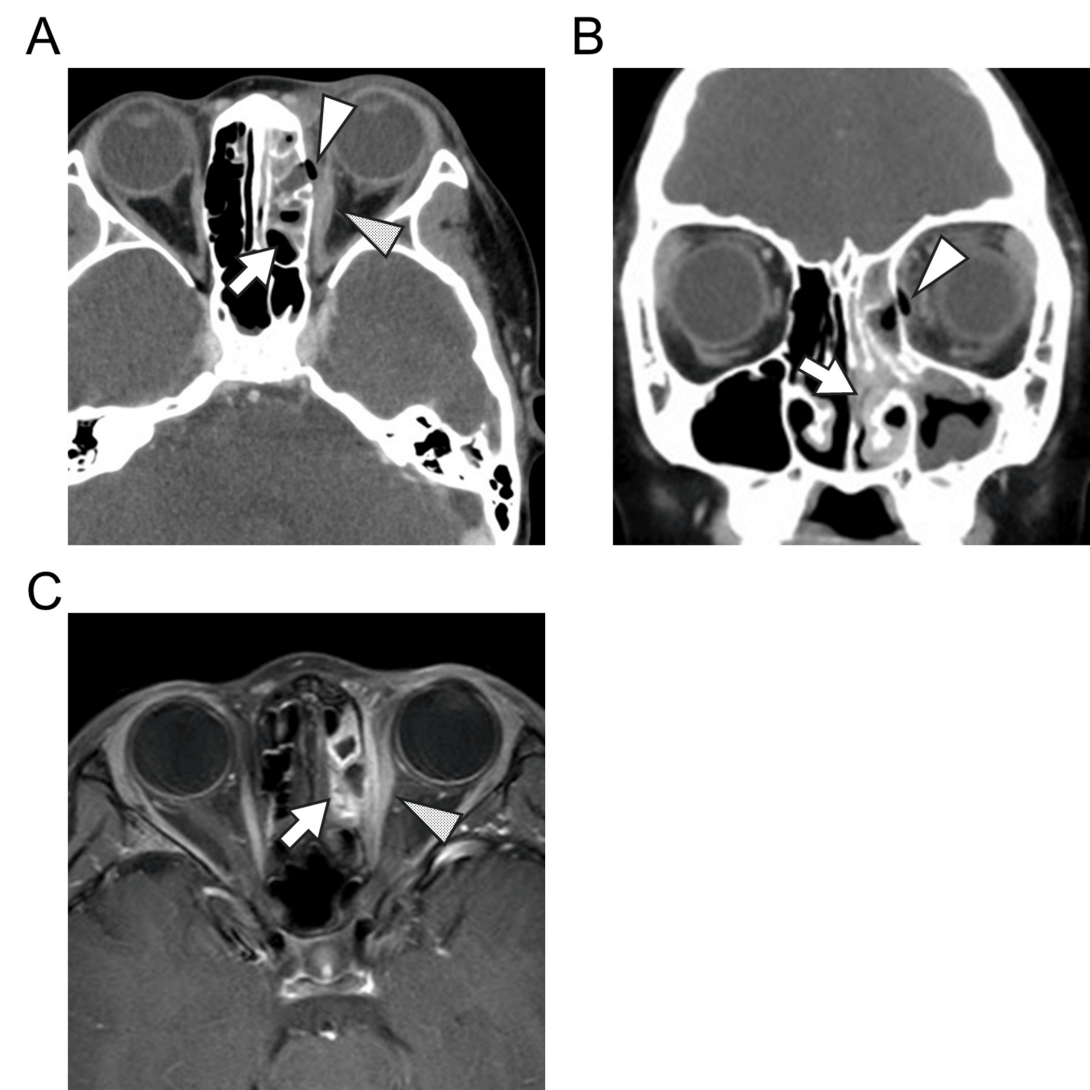
Imaging studies of the patient showing left orbital cellulitis (A) Axial contrast-enhanced CT of the orbit on admission. Cellulitis of the eyelid extended to the cheek. Sinusitis (arrow), air in the orbit (white arrowhead), and mild thickening of the medial rectus muscle (dotted arrowhead) are noted on the left side. (B) Coronal contrast-enhanced CT of the orbit on admission. Sinusitis (arrow) and air in the orbit (white arrowhead) are noted on the left side. (C) Contrast-enhanced T1-weighted MRI of the orbit (axial view). Residual left sinusitis (arrow) is observed. The contrast effect is noted in the left medial rectus muscle (dotted arrowhead). No abscess is observed in the orbit.

Contrast-enhanced CT of the orbit revealed left-sided sinusitis with fat stranding around the left nasolacrimal duct, suggesting a potential primary focus of infection. There was also cellulitis extending from the left eyelid to the cheek. In addition, a partial defect of the lamina papyracea in the left ethmoid bone was suspected, along with a small air space in the orbit (Figure 1 A, B). No abscess was observed in the orbit. Mild swelling of the left medial rectus muscle was noted, raising debate over whether this represented a true finding of orbital cellulitis or was merely an artifact. However, the presence of air within the orbit suggested that the inflammation had extended posterior to the orbital septum, reaching the lateral aspect of the left globe, leading to a definitive diagnosis of orbital cellulitis.

Considering the presence of air within the orbit, the possibility of infection with gas-producing bacteria was also taken into account. Therefore, in view of the potential involvement of GAS, anaerobic bacteria, and methicillin-resistant *Staphylococcus aureus* (MRSA), combination therapy was initiated with cefotaxime (300 mg/kg/day), clindamycin (900 mg/day), and vancomycin (1500 mg/day). Two days later, the patient became afebrile, and inflammation around the left eye improved, allowing eye opening.

Aerobic cultures of nasal secretions and ocular discharge obtained at admission detected *Moraxella catarrhalis*, which was susceptible to antibiotics. As *M. catarrhalis* has been linked to orbital cellulitis with abscess formation, an MRI performed on hospital day 7 confirmed the absence of an abscess (Figure 1C). On day 9, antibiotics were switched to oral amoxicillin/clavulanic acid (250 mg, four times daily) for 14 days. With a favorable clinical course, treatment was discontinued. Blood cultures obtained at admission were negative.

## Discussion

This case involved a seven-year-old girl who presented with fever, headache, and subsequent development of left retro-orbital pain and photophobia. Although these symptoms can be associated with sinusitis, the rapid progression of periorbital swelling raised concern for orbital cellulitis. Despite the absence of obvious proptosis or visual impairment, contrast-enhanced CT was performed to evaluate possible orbital involvement. Imaging revealed mild orbital inflammation and intraorbital gas, leading to the diagnosis. Although no intraorbital cultures were obtained, broad-spectrum antibiotic therapy was initiated to cover a wide range of potential pathogens, resulting in full recovery without sequelae.

In this case, the time course of symptoms, including retro-orbital pain and photophobia, raised clinical suspicion for orbital cellulitis. However, periorbital cellulitis was also considered in the differential diagnosis, as ocular motility was preserved and there were no signs of proptosis or visual impairment. Typically, periorbital cellulitis lacks proptosis, restricted motility, or vision loss. However, studies report that 20-25% of pediatric orbital cellulitis cases present without proptosis, underscoring the need for imaging in suspected cases. Historically, untreated orbital cellulitis led to blindness in 20% of cases and death in 17% due to central nervous system complications [8], emphasizing its severity.

According to the *Guidelines for the Management of Periorbital Cellulitis/Abscess* [2], contrast-enhanced CT imaging should be considered when any of the following are present: central signs, inability to accurately assess vision, gross proptosis, ophthalmoplegia, deteriorating visual acuity or color vision, bilateral periorbital edema, no improvement or clinical deterioration within 24 hours, or swinging pyrexia persisting beyond 36 hours. Although our patient did not meet these criteria, the combination of her presenting symptoms and their rapid progression raised clinical suspicion for orbital cellulitis, prompting early imaging and diagnosis. It may be beneficial to consider both the type and progression of symptoms in conjunction with these criteria when determining the need for CT imaging.

In this case, the absence of proptosis, the lack of abscess formation, and only mild thickening of the medial rectus muscle made the differential diagnosis challenging. However, the presence of intraorbital gas was a crucial factor in diagnosing orbital cellulitis. Cases with intraorbital gas often require surgical drainage due to concerns about gas-producing bacterial infections, making resolution with antibiotics alone a notable finding. Early recognition of orbital cellulitis and prompt antibiotic therapy likely contributed to the favorable outcome.

Two mechanisms may explain the intraorbital gas in this case. One possibility is bacterial gas production, as many reported cases involve anaerobic infections. Although anaerobic involvement was not confirmed, the use of broad-spectrum antibiotics with anaerobic coverage may have contributed to the patient's recovery. Another possibility is air leakage from the paranasal sinuses. Contrast-enhanced CT revealed severe left-sided sinusitis and a small lamina papyracea defect, suggesting that sinusitis-induced erosion led to air entry into the orbit. Defects in the lamina papyracea, either congenital or sinusitis-related, are reported in 0.5%-10% of non-traumatic cases [9]. Pediatric patients are particularly susceptible due to thin, underdeveloped bones and unfused cranial sutures, facilitating the spread of inflammation and air leakage [10,11].

Identifying the causative pathogen in orbital cellulitis is often challenging without surgical drainage. In this case, contrast-enhanced CT suggested infection spread from the paranasal sinuses, implicating sinusitis pathogens. *M. catarrhalis*, isolated from nasal and ocular samples, is a potential causative organism. Anaerobic bacteria, frequently detected in pediatric sinusitis [12], could also be involved, given the presence of intraorbital gas. Therefore, in this case, the possibility of anaerobic infection was considered when selecting the initial antibiotic regimen. The finding of intraorbital gas was not only crucial for diagnosis but also played a significant role in guiding treatment choices. Additionally, GAS was detected in the pharynx, suggesting a possible contribution. Since precise pathogen identification is difficult without drainage, careful evaluation of clinical symptoms and progression is crucial for selecting appropriate antibiotics.

## Conclusions

A seven-year-old girl presented with periorbital inflammation and fever. Differentiating orbital cellulitis from periorbital cellulitis based solely on physical examination was challenging. Based on the patient's symptoms and their clinical progression, contrast-enhanced CT was considered and proved invaluable for diagnosis, with intraorbital air serving as a particularly specific finding. Given the potential for severe complications, prompt diagnosis and intervention are crucial in cases of orbital cellulitis. When differentiation between these conditions is difficult, contrast-enhanced CT can be a useful tool to aid in diagnosis.
